# Pamphlets Received

**Published:** 1881-02

**Authors:** 


PAMPHLETS RECEIVED.—The Development of the Osseous Callus in Fractures of the Bones in Man and Animals. By Henry O. Marcy, A.M., M.D., Member of Am. Med. Association. Reprint.

"The Physician's and Surgeon's Investigator." A new monthly journal published under the auspices of the College of Physicians and Surgeons, Buffalo, N. Y., and edited by A. A. Hubbell, M.D.

Vick's Floral Guide for 1881. One of the best gotten up floral catalogues and descriptive lists we have ever seen.

Cæsarian Section with Removal of Uterus and Ovaries after the Porro-Muller Method. By Elliott Richardson, M.D., Lecturer on Practical Obstetrics, University of Pennsylvania, etc, Reprint from *Am. Jour. Med. Sciences*, January, 1881. Complimentary.

